# GaInNAs-based Hellish-vertical cavity semiconductor optical amplifier for 1.3 μm operation

**DOI:** 10.1186/1556-276X-6-104

**Published:** 2011-01-27

**Authors:** Faten Adel Ismail Chaqmaqchee, Simone Mazzucato, Murat Oduncuoglu, Naci Balkan, Yun Sun, Mustafa Gunes, Maxime Hugues, Mark Hopkinson

**Affiliations:** 1 School of Computer Science and Electronic Engineering, University of Essex, Colchester CO4 3SQ, UK; 2 Department of Physics, Faculty of Science and Art, University of Kilis 7 Aralik, Kilis, Turkey; 3 Department of Electronic and Electrical Engineering, University of Sheffield, Sheffield S1 3JD, UK

## Abstract

Hot electron light emission and lasing in semiconductor heterostructure (Hellish) devices are surface emitters the operation of which is based on the longitudinal injection of electrons and holes in the active region. These devices can be designed to be used as vertical cavity surface emitting laser or, as in this study, as a vertical cavity semiconductor optical amplifier (VCSOA). This study investigates the prospects for a Hellish VCSOA based on GaInNAs/GaAs material for operation in the 1.3-μm wavelength range. Hellish VCSOAs have increased functionality, and use undoped distributed Bragg reflectors; and this coupled with direct injection into the active region is expected to yield improvements in the gain and bandwidth. The design of the Hellish VCSOA is based on the transfer matrix method and the optical field distribution within the structure, where the determination of the position of quantum wells is crucial. A full assessment of Hellish VCSOAs has been performed in a device with eleven layers of Ga_0.35_In_0.65_N_0.02_As_0.08_/GaAs quantum wells (QWs) in the active region. It was characterised through *I*-*V*, *L*-*V *and by spectral photoluminescence, electroluminescence and electro-photoluminescence as a function of temperature and applied bias. Cavity resonance and gain peak curves have been calculated at different temperatures. Good agreement between experimental and theoretical results has been obtained.

## Introduction

III-V semiconductors are indispensable for today's optoelectronic devices, such as lasers modulators, photodetectors and optical amplifiers in optical fibre communication systems. One potentially important material for such applications is the quaternary alloy GaInNAs [1,2]. In the 1.3-μm optical communications window, GaInNAs may be grown pseudomorphically on GaAs, allowing the use of high quality AlAs/GaAs distributed Bragg reflectors (DBRs), with potential cost advantages compared to InP-based approaches. It can be used to fabricate several devices, among which vertical cavity semiconductor optical amplifiers (VCSOAs) are important components in optical fibre networks. They have improved performance over SOAs as they have inherent polarization insensitivity, lower noise figures, high-fibre coupling, easy chip testing and potential for integration into high-density two-dimensional arrays. Furthermore the narrower bandwidth of vertical cavity structures makes these devices good for filtering applications [3-6].

A VCSOA can be simply described as a vertical cavity surface emitting laser (VCSEL) operating in the linear regime below threshold, with a reduced number of top DBR layers. However, in this article, a novel VCSOA based on the Hellish structure as an alternative to conventional VCSOAs is investigated [7]. Hellish devices utilise the transport of non-equilibrium carriers parallel to the layers. Spontaneous emission of ultra bright Hellish structures has been demonstrated [8,9]. VCSEL operation was achieved by addiction of DBR layers [10-13]. That design is adapted in this study to make a GaInNAs-based Hellish-VCSOA structure, which differs from the conventional VCSEL by the reduced number of top DBR layers [14]. The structure is designed to operate in the 1.3-μm wavelength region via electrical pumping.

The authors demonstrate for the first time the operation of a Hellish VCSOA with a multiple quantum well (MQW) GaInNAs/GaAs active region, at temperatures between 77 and 300 K. Optical and electrical pumping (photoluminescence—PL, electroluminescence—EL) were used, and a 1.28-μm emission at room temperature was observed. By combining the two measurements, an electro-photoluminescence (EPL) technique was performed, from which light amplification is demonstrated. The authors also present the results of the reflectivity spectrum and cavity resonance calculations using the matrix formulation for multi-layer structures [15], and compare these with experimental results.

### Experimental results and discussion

**Figure 1:**
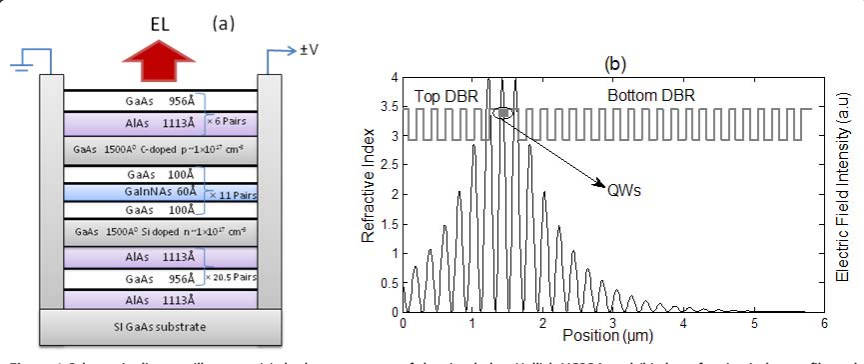
Schematic diagram illustrates (a) the layer structure of the simple bar Hellish-VCSOA and (b) the refractive index profile and distribution of the electric filed intensity across the sample, in which the QWs are situated at the antinode of the electric field, i.e. where it reaches its maximum intensity.

The structure of the investigated device, shown in Figure 1a, contains 11 layers of 6 nm-thick Ga_0.35_In_0.65_N_0.02_As_0.08 _quantum wells separated by 10 nm GaAs barriers. The use of MQWs, placed at the electric field antinode of 3*λ*/2 cavity length, is done in order to provide optical gain (Figure 1b). The active region is enclosed between two 150 nm-thick doped cladding layers Si-doped (*n *= 1 × 10^17 ^cm^-3^) on the bottom side, and C-doped (*p *= 1 × 10^17 ^cm^-3^) on the top side. The structure is sandwiched between two DBRs. The bottom DBR has 20.5 pairs of AlAs/GaAs quarter-wave stacks and provides a reflectivity in excess of 99% at 1.3-μm. On the other side, the top DBR has six pairs of AlAs/GaAs quarter-wave stacks giving around 60% reflectivity. This is lower than the bottom DBR, thus allowing light emission from the top surface. Both DBRs are undoped except for the first bottom AlAs/GaAs period which is 1 × 10^17 ^cm^-3 ^doped.

**Figure 2:**
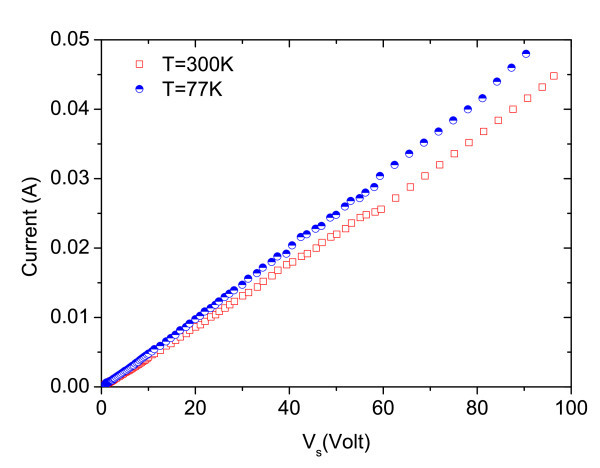
***I*-*V *characteristics of simple bar Hellish-VCSOA at 77 and 300 K **.

Ohmic contacts are formed by diffusing Au/GeAu/Ni/Au through all the layers and into the substrate, defining a simple bar-shaped sample, with 1-mm contact separation and 4.5-mm width. This is done by annealing the contacts for 60 s at 430°C. Once fabricated, the device is electrically biased with positive voltage pulses 390-ns duration and a 3-ms repetition rate. The duty cycle is small enough to prevent damage by excessive Joule heating. The applied electric field is varied from 0.01 to 1 kV/cm. Figure 2 shows the current-voltage (*I*-*V*) characteristics at 77 and 300 K. The sample exhibits ohmic behaviour at electric fields below 600 and 900 V/cm at 77 and 300 K, respectively. The small deviation from ohmic behaviour is an indication of carrier heating [16,17].

**Figure 3:**
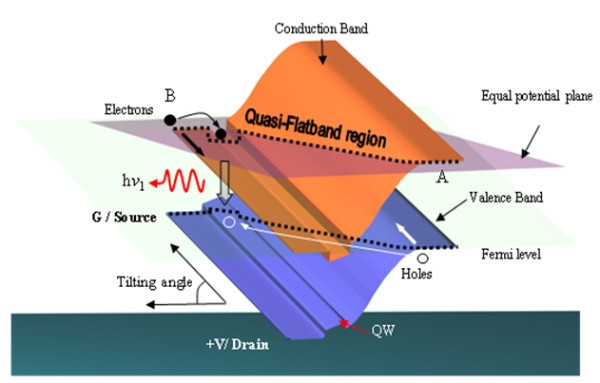
**Schematic diagram to illustrate the emission of light under quasi-flat band region condition **[16].

The operation of Hellish device is based on the longitudinal injection of electron and hole pairs in their respective channels, due to the diffusion of both top contacts through all layers. Without the applied electric field, if the sample is illuminated, photogenerated carriers will eventually recombine radiatively in the QW without drifting laterally in the longitudinal channels. On the other hand, when the device is biased, the energy bands tilt up, with the degree of tilting being proportional to the applied voltage. At low bias, a quasi-flat region is established by the tilted energy bands, and a small number of carriers are then able to drift diagonally into the *p*-*n *junction. This is illustrated in Figure 3. With an increase in the electric field, the energy bands will tilt up more, so that more carriers will flow into the active region, enhancing the emitted light. In view of the operational diagram depicted in Figure 3, the application of a negative bias results in a tilting and the diffusion of the holes to the region where electrons are injected, and recombination occurs in the vicinity of the cathode. This allows for spatial confinement and control of the light emission area. Luminescence from the opposite site (anode) appears by inverting the bias polarity [16].

**Figure 4:**
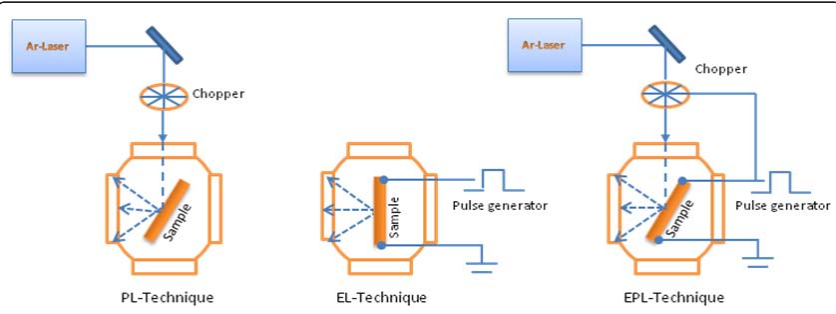
**PL, EL and EPL experimental arrangement**.

Experiments have been carried out using PL, EL and EPL techniques at different temperatures between liquid nitrogen and room temperature. The experimental arrangement for these techniques is illustrated in Figure 4.

In PL and EPL, the optical excitation source is a CW Argon laser operating at 488-nm wavelength with 20-mW output power. The laser beam is chopped using a mechanical chopper and directed to the sample surface. The emitted light is dispersed by a Bentham M300 1/3 m monochromator and collected with a cooled InGaAs photomultiplier. The outcoming electrical signal is sent to a Gated Integrator & Boxcar Averager Module (Stanford Research Systems, model SR250) or a lock-in amplifier (Stanford Research Systems, model SR830) according to the experiment performed.

**Figure 5:**
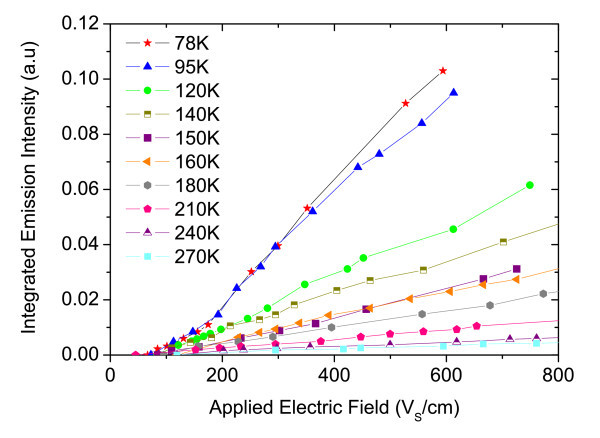
**Integrated EL intensity versus applied electric field at various temperatures**.

Figure 5 shows the integrated emission light from the device as a function of applied electric field. The threshold light emission varies between 110 and 290 V/cm according to the sample temperature. Above the threshold, the integrated EL increases linearly with applied electric field.

**Figure 6:**
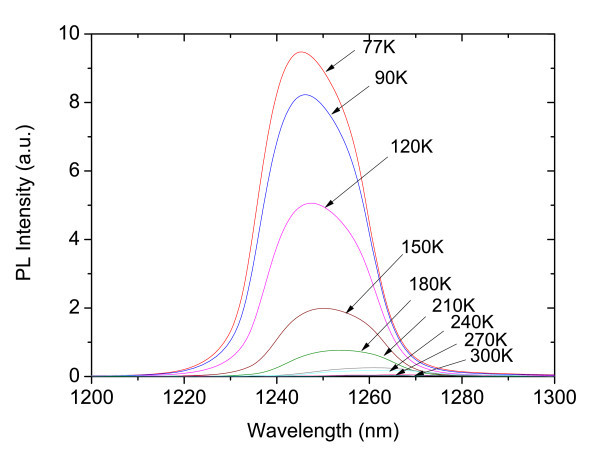
**PL spectra measured at different temperatures**.

Figure 6 shows the PL spectra measured at different temperatures. The PL peak red-shifts from 1245 nm at 77 K to 1270 nm at 300 K.

**Figure 7:**
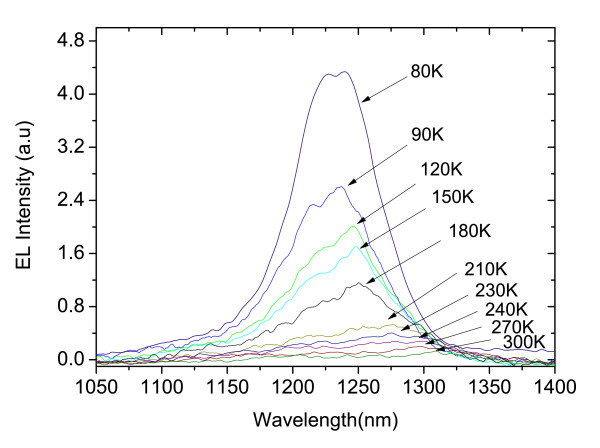
**EL spectra measured at a fixed bias voltage of 97 V corresponding to an electric field of 0.97 kV/cm**.

Spectral EL is also measured with applied voltage pulses of amplitude between 0.3 and 100 V, where the pulse duration is kept at about 390 ns. The EL spectra are obtained at different temperatures between 80 and 300 K, and according to Figure 7, it shows a broad spectra. Approximately, the EL spectrum shifts in wavelength from 1239 nm at 80 K to 1281 nm at 300 K.

**Figure 8:**
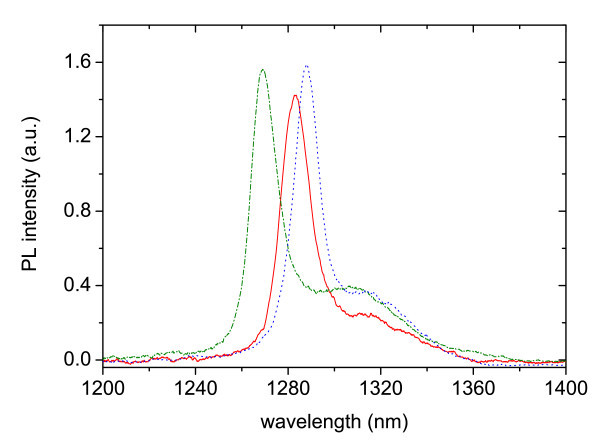
**Room temperature PL spectra taken at different laser spot positions across the sample, showing an approximate 20-nm uncertainty in the peak position**.

**Figure 9:**
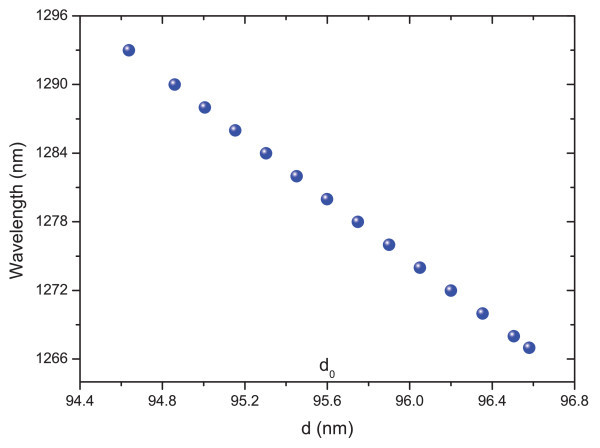
Dependence of the cavity resonance position with ±1 nm fluctuations of the GaAs thickness in the DBR (d_0_ is the nominal GaAs DBR layer thickness) but weaker behaviour takes place when fluctuations occur in the AlAs layers.

There is good agreement between the EL and PL peak positions. However, the EL emission is considerably broader than the PL. This observation is attributed to growth non-uniformities and material fluctuations. PL is measured from a small spot (excitation spot size 0.5 mm^2^), while the EL is collected from the whole sample surface. Therefore, the EL may be expected to be broader if the QWs and/or DBRs width have monolayer fluctuations. In order to prove this, the PL at different spots on the sample (Figure 8) was measured and the reflectivity spectrum for small fluctuations in the thickness of the layers in the cavity of around 2 nm (Figure 9) was calculated. The effect of layer fluctuations is clear.

**Figure 10:**
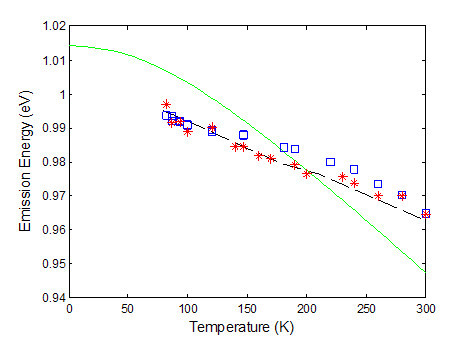
Continuous line represents the calculated temperature dependence of bandgap energy for the device active area (GaInNAs/GaAs QW) using the BAC model, while the expected cavity resonance position is plotted with a dashed line and finally the scattered points represent the experimental data for PL (asterisks) and EL (squares).

The temperature dependence of EL and PL peaks and the cavity resonance are plotted in Figure 10, together with the active material bandgap energy curve [18]. The behaviour differs extremely from the change of the GaInNAs/GaAs bandgap energy with temperature. Theoretically, a red shift of the active material peak wavelength at a rate of 0.38 nm/K was predicted, while the resonance cavity moves with temperature at 0.18 nm/K. At these rates, the optimum operating temperature for this device will be at around 220 K, where the maximum peak material gain coincides with the DBR resonance cavity position.

The EPL technique was performed by combining the two experimental techniques, namely PL and EL. In order to synchronise optical and electrical pulses, the pulse generator is triggered by a mechanical chopper. PL, EL and EPL spectra for Hellish-VCSOA are measured as a function of temperature. In both EL and EPL, the electric field was kept constant at 0.7 kV/cm.

**Figure 11:**
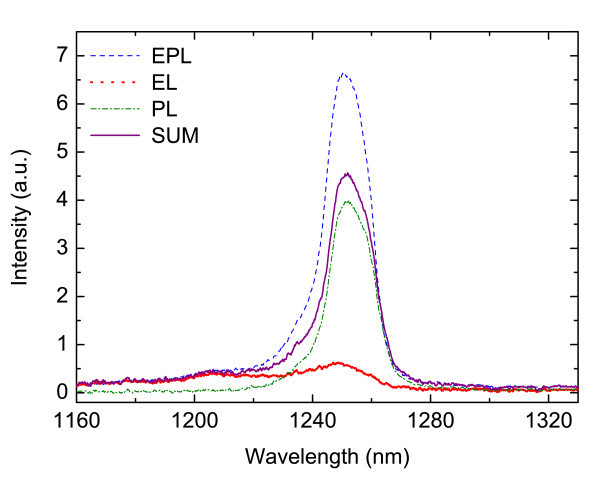
**EL, PL, EPL and SUM (EL + PL) spectra at *T *= 87 K**.

In Figure 11, the *T *= 87 K EL, PL, EPL spectra and the sum of EL and PL are plotted. The PL spectrum presents a broad peak at around 1250-nm wavelength and a full-width-at-half-maximum of 13 meV. As stated before, it corresponds to the overlap of the active region gain spectrum and the cavity resonance reflectivity that filters and narrows the emission. Variations in the peak position are ascribable to fluctuations in the cavity resonance. The EL spectrum measured at the same temperature shows the emission peak at around 1.03 eV and by comparing the SUM (EL + PL) and EPL spectrum, the presence of optical gain was clearly visible. Signal amplification occurs when both electrical and optical inputs are applied.

**Figure 12:**
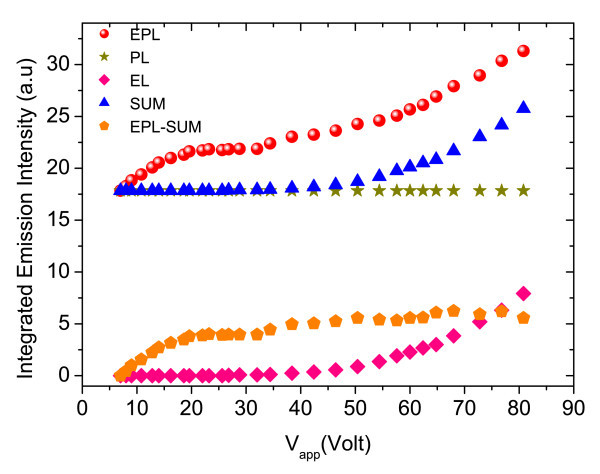
**Integrated EPL, PL, EL, SUM (EL + PL) and EPL-SUM (EL + PL) measured as a function of applied voltages at *T *= 300 K**.

**Figure 13:**
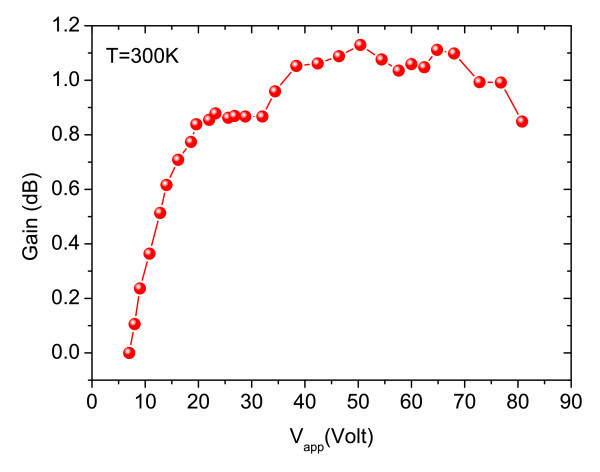
**Gain characteristics are measured as a function of applied voltages at *T *= 300 K**.

This investigation was focussed on the gain at room temperature. The integrated intensities of PL, EL and EPL, together with the calculated SUM (EL + PL) and gain are plotted in Figure 12, as function of applied voltage, up to 800 V/cm, with laser excitation power of 10 mW. Finally, Figure 13 displays the evolution of the gain with applied voltage, which reaches its maximum at around 50 V. It should be noted that the wavelength of the laser (*λ *= 488 nm) is very different from the cavity resonance position shown in Figure 10. Therefore, most of the excitation is lost through absorption. In order to give a quantitative value to the VCSOA gain, the PL gain is defined as the ratio of the PL peak when the device is electrically pumped to that when the device is not biased. This gain should not be confused with conventional VCSOA gain as ratio of output power to input power.

Further improvements in gain characteristic and device performance will be expected by optimising the Hellish-VCSOA structure for 1.3-μm application via electrically pumping, and by reducing the device length so that the operating voltage will be much lower than the one used here.

## Conclusions

Optical gain at *λ *~ 1.28 μm is demonstrated in a Hellish-VCSOA device consisting of Ga_0.35_In_0.65_N_0.02_As_0.08_/GaAs QWs and AlAs/GaAs DBRs. The advantage of using such device is that longitudinal electric fields are applied parallel to active layer so that the current flows along the *p *and *n *layers without passing through the DBRs. The operation of the device is independent of the polarity of the applied electric field. The emission and amplification characteristics are investigated as a function of temperature and applied voltage. Thus, the Hellish-VCSOA is a good candidate for electrically pumped optical amplifier operating at around 1.3 μm.

## Abbreviations

DBRs: distributed Bragg reflectors; EL: electroluminescence; EPL: electro-photoluminescence; MQWs: multiple quantum wells; PL: photoluminescence; QWs: quantum wells; VCSEL: vertical cavity surface emitting laser; VCSOA: vertical cavity semiconductor optical amplifier.

## Competing interests

The authors declare that they have no competing interests.

## Authors' contributions

FAI, YS and MO designed the structure. MHu and MHo grew the sample according to the specifications. FAI fabricated the devices, carried out the experiments and the theoretical calculations, in collaboration with MO, YS, SM, and MG. FAI and SM wrote up the article. NB, is the inventor of the original device and the overall supervisor of the project.

All authors read and approved the final manuscript.
